# Active control on high-order coherence and statistic characterization on random phase fluctuation of two classical point sources

**DOI:** 10.1038/srep23614

**Published:** 2016-03-29

**Authors:** Peilong Hong, Liming Li, Jianji Liu, Guoquan Zhang

**Affiliations:** 1 The MOE Key Laboratory of Weak Light Nonlinear Photonics, School of Physics and TEDA Applied Physics Institute, Nankai University, Tianjin 300457, China

## Abstract

Young’s double-slit or two-beam interference is of fundamental importance to understand various interference effects, in which the stationary phase difference between two beams plays the key role in the first-order coherence. Different from the case of first-order coherence, in the high-order optical coherence the statistic behavior of the optical phase will play the key role. In this article, by employing a fundamental interfering configuration with two classical point sources, we showed that the high- order optical coherence between two classical point sources can be actively designed by controlling the statistic behavior of the relative phase difference between two point sources. Synchronous position *N*th-order subwavelength interference with an effective wavelength of *λ*/*M* was demonstrated, in which *λ* is the wavelength of point sources and *M* is an integer not larger than *N*. Interestingly, we found that the synchronous position *N*th-order interference fringe fingerprints the statistic trace of random phase fluctuation of two classical point sources, therefore, it provides an effective way to characterize the statistic properties of phase fluctuation for incoherent light sources.

Optical high-order coherence effect was first reported by Hanbury Brown and Twiss (HBT) in 1956, where interference among randomly distributed first-order incoherent point emitters forms the bunching effect of a thermal light source in the far-field plane^1^,^2^. Since then, much attention has been paid to the field of optical high-order coherence, leading to discovery of many intriguing interference effects^3^,^4^,^5^,^6^ and applications such as ghost imaging^7^,^8^,^9^,^10^, subwavelength interference and optical lithography^6^,^11^,^12^,^13^,^14^,^15^,^16^, and super-resolving measurements^4^,^17^,^18^,^19^,^20^.

In the field of optical coherence, one of the most important fundamental issues is two-beam interference, which is the basis to understand various interference effects observed in various interferometers, and it can be historically traced back to Young’s double-slit interference, where the stationary phase difference between two beams plays the key role in the first-order coherence^21^. High-order optical coherence with various light sources^22^,^23^,^24^,^25^,^26^,^27^,^28^ was extensively studied, and much attention was paid to the quantum or classical properties of light sources, offering the basic understanding of many novel high-order coherence effects^4^,^5^,^6^. In contrast to the case of first-order coherence, although high-order coherence of two completely independent light sources are well studied and understood^23^,^24^, effects of optical phase on the high-order optical coherence are largely ignored. Interestingly, recent progresses on high-order coherence of classical light show that modulation on the wavefront of classical light sources will generate novel interference effects such as subwavelength interference^16^,^17^,^29^,^30^,^31^,^32^ and non-Rayleigh speckles with tailored intensity statistics^33^, indicating the importance of optical phase in high-order optical coherence.

In this article, we studied the effects of optical phase on high-order optical coherence in a fundamental interfering configuration with two classical point sources. We showed that, different from the case in the first-order optical coherence where a stationary phase difference between two beams is important, in high-order optical coherence the statistic behavior of the optical phase difference between light beams will play an important role. Therefore, it is possible for one to actively design the high-order optical coherence for particular applications such as subwavelength interference and high spatial resolution optical lithography through control on the statistic behavior of optical phase of light sources. Furthermore, we found that the high-order optical interference pattern reveals the statistic properties of the relative phase fluctuation of interfering light sources, which is of fundamental importance in optical science.

## Results

### Theoretical Analysis

**Figure 1:**
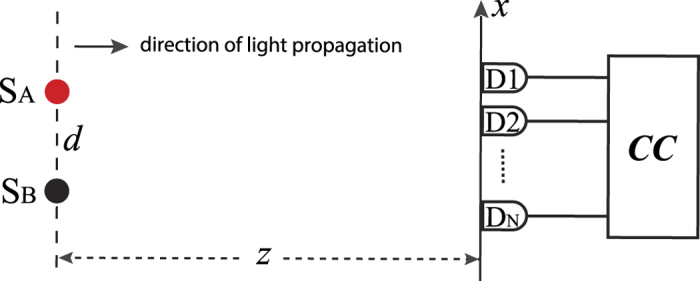
Fundamental scheme to study the first- and high-order coherence between two classical point sources *S*_*A*_ and *S*_*B*_. *D*_*i*_: photodetector. *CC*: coincidence count.

Figure 1 shows the fundamental scheme to study the first and high-order coherence effects between two spatially separated classical point sources *S*_*A*_ and *S*_*B*_, in which *D*_*i*_ 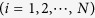 is single-photon detector in the far-field observation plane, *d* is the distance between *S*_*A*_ and *S*_*B*_, and *z* is the distance between the observation plane and the source plane, respectively. When the relative phase difference *φ* between the two point sources is stationary, they are first-order coherent with respect to each other, and the scheme is essentially a Young’s double-slit interference scheme, which will show an intensity interference fringe *I*(*x*) ∝ 1 + cos(*kdx*/*z*) with *k* being the wave number and *x* being the observation coordinate in the far-field plane. However, due to the complimentary effect between the first-order coherence and the high-order coherence^23^,^24^,^34^, no high-order interference fringe can be observed in this case. In the other extreme case, when the two point sources are completely independent and the relative phase difference *φ* varies randomly in the range [0, 2*π*), it is evident that the two point sources are first-order incoherent, but one can observe high-order interference fringes, for example, two-photon interference fringe 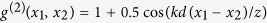 with respect to the spatial separation between two observation points (*x*_1_ − *x*_2_). One may already notice that the relative phase difference *φ* plays an important role in not only the first-order coherence but also the high-order coherence. In the following, we will show that the high-order coherence properties can be actively designed by controlling the statistic behavior of the relative phase difference *φ*.

**Figure 2:**
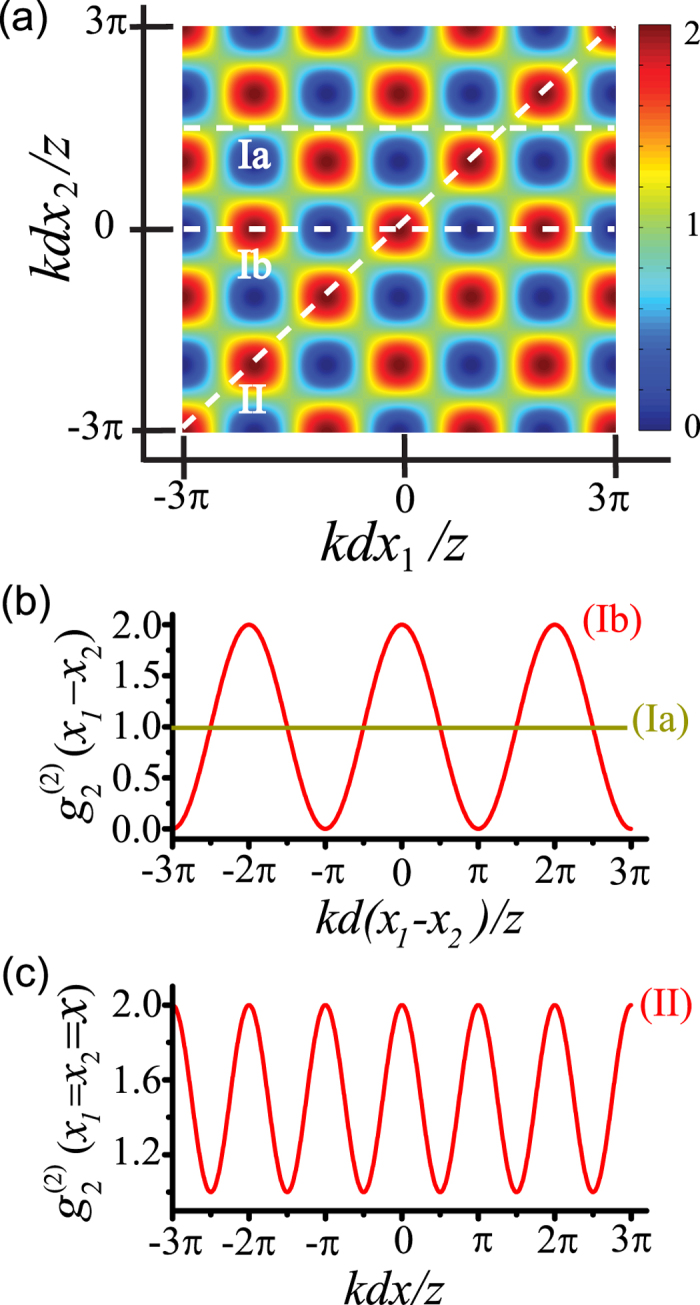
Two-dimension second-order coherence pattern (**a**) when the relative phase difference *φ* varies randomly between {0, *π*} with equal probability 0.5. (**b**) depicts the two-photon interference fringes by scanning the detectors along lines Ia and Ib, respectively. (**c**) shows synchronous position subwavelength two-photon interference when scanning the detectors along line II.

Let’s first consider the simplest special case when the relative phase difference *φ* varies randomly between two critical values {*θ*_1_, *θ*_2_} with corresponding probability *P*_1_ and *P*_2_, respectively. According to the complimentary rule, the high-order interference fringes can be observed when the intensity interference fringes between *S*_*A*_ and *S*_*B*_ are completely washed out, i.e.,


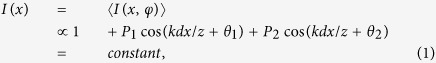


where 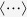 means an ensemble average. This is satisfied when one sets *θ*_2_ − *θ*_1_ = *π* and *P*_1_ = *P*_2_ = 0.5. Under this condition, the normalized second-order intensity correlation function can be calculated as


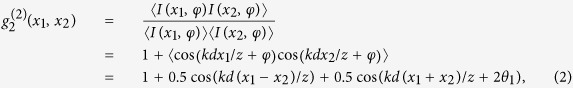


where the subscript “2” in 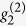 indicates the number of the critical phase *θ*_*i*_ . One sees that the second-order interference pattern is completely different from that discussed previously. Besides the well-known cosine fringe cos(*kd*(*x*_1_ − *x*_2_)/*z*) with respect to (*x*_1_ − *x*_2_), an additional set of cosine fringe cos(*kd*(*x*_1 _ − *x*_2_)/*z* + 2*θ*_1_) with respect to (*x*_1_ + *x*_2_) appears. The second-order interference pattern is the sum of these two kinds of interference fringes, which makes the total interference pattern quite richer as shown in Fig. 2. One notes that the second-order interference fringe is dependent on both the positions and the scanning mode of two photodetectors. For example, no second-order interference fringe can be observed when one fixes detector *D*_2_ and scans detector *D*_1_ along line Ia in Fig. 2(a), while a periodical cosine fringe with a visibility of 100%, which is usually thought as a property of two-photon interference with quantum single-photon sources^23^,^24^, is obtained when one scans the detector *D*_1_ along line Ib (see Fig. 2(b)). More interestingly, when one sets the two detectors at exactly the same position *x*_1_ = *x*_2_ = *x* and scans them together along line II, synchronous position subwavelength two-photon interference fringe can be observed with an effective wavelength being half of the original wavelength of the point sources (see Fig. 2(c)). Such synchronous position subwavelength interference is helpful to improve the resolution of optical lithography^11^,^12^.

Furthermore, the *N*th-order coherence can be described by the normalized *N*th-order intensity correlation function


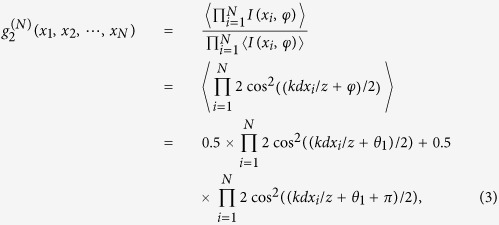


which shows very complicated interference fringes with respect to the position of each space point *x*_*i*_. For practical applications such as multi-photon lithography and holography, we hereafter will only consider the synchronous position *N*th-order correlation function with 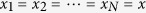, and it evolves to


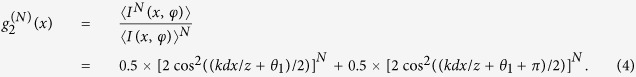


Two elementary fringes in the form of 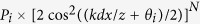 are included in Eq. (4), which are phase-shifted by *π* with respect to each other. One sees that, it is the sum of these two elementary fringes that results in subwavelength interference with an effective wavelength of *λ*/2. Furthermore, the visibility of the interference fringes grows with *N*. Interestingly, the high-order interference fringe (*N* ≥ 2) reveals the statistic fluctuation of the relative phase difference *φ* between *S*_*A*_ and *S*_*B*_. From Eq. (4), one can see that the peak amplitude and the peak position of the interference fringe are determined by the probability *P*_*i*_ and the critical phase value *θ*_*i*_, respectively. Therefore, one can extract the statistic information of the phase variation *φ* between two point sources such as the critical relative phase value *θ*_*i*_ and its probability *P*_*i*_ simply by measuring the synchronous position *N*th-order correlation function. Although optical noise may also be amplified during the correlation treatment, one should note that the signal is amplified much more as compared to optical noise, therefore, the signal-to-noise ratio and the contrast-to-noise ratio will be improved in high-order correlation measurements, which was well verified both experimentally and theoretically^35^,^36^.

When the relative phase difference *φ* varies randomly among three critical values {*θ*_1_, *θ*_2_, *θ*_3_}, each with a probability *P*_1_, *P*_2_ and *P*_3_, respectively. Under the condition that


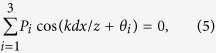


the first-order interference fringe between *S*_*A*_ and *S*_*B*_ is washed out, and the normalized synchronous position *N*th-order interference fringes can be described as


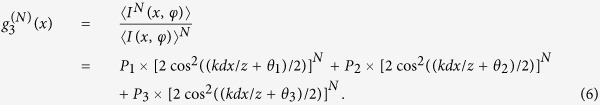


**Figure 3:**
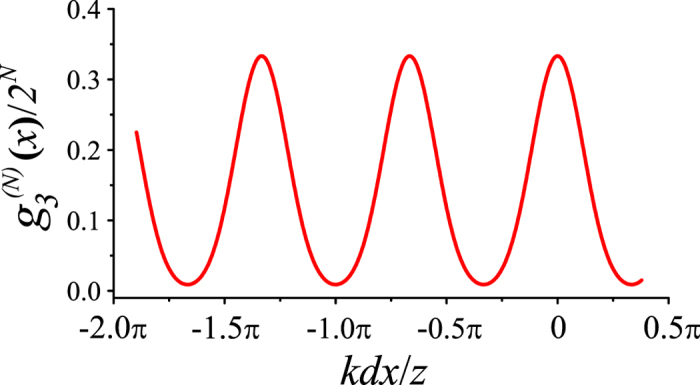
Synchronous position subwavelength interference pattern when the relative phase difference *φ* varies randomly among {0, −2*π*/3, −4*π*/3} with equal probability 1/3. Here *N* is set to be 15.

Similar to the case of 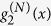 in Eq. (4), three elementary fringes in the formula 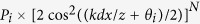 are included in Eq. (6). Again, one can see that, the fringe peak amplitude is determined by the probability *P*_*i*_ and the peak position is determined by the critical phase value *θ*_*i*_. Eq. (5) can be satisfied with various sets of {*P*_*i*_, *θ*_*i*_} (see Methods for the general way to find out the solution {*P*_*i*_, *θ*_*i*_}), therefore, various interference fringes can be actively designed according to the requirement of practical applications. For example, by setting the relative phase difference *φ* to vary randomly among {0, −2*π*/3, −4*π*/3} with equal probability 1/3, one can get a set of synchronous position *N*th-order interference fringe (*N* ≥ 3) with an effective wavelength of *λ*/3, where *λ* is the original wavelength of the point source, as shown in Fig. 3. On the other hand, one can get the statistic variation information (*P*_*i*_, *θ*_*i*_) of the relative phase difference *φ* simply by measuring the synchronous position high-order interference fringes, as we will demonstrate experimentally in the following section. Note that, to clearly show the relationship between the peak amplitude and position of the interference fringe and the statistic parameters (*P*_*i*_, *θ*_*i*_) of the relative phase difference *φ*, the vertical axis of Fig. 3 is scaled by a factor 2^*N*^.

### Experimental verification

**Figure 4:**
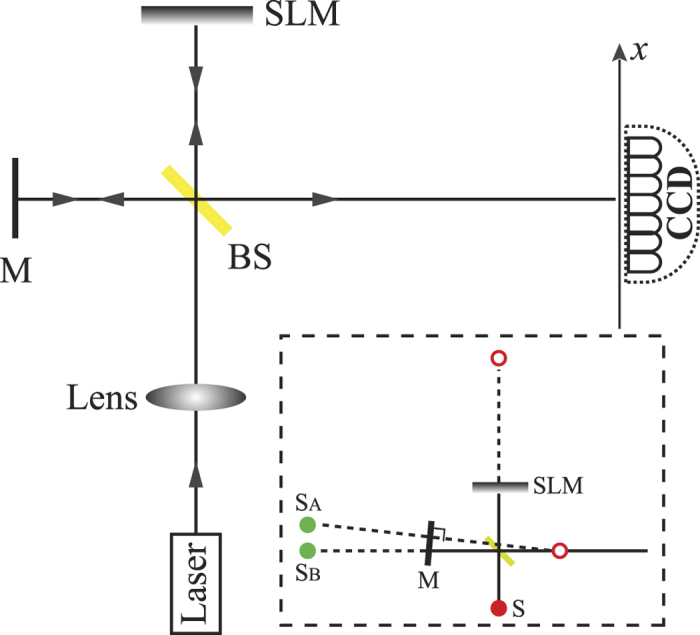
The schematic diagram of the experimental setup to measure the high-order optical coherence of two classical point sources. The inset shows the generation of two effective point sources *S*_*A*_ and *S*_*B*_ by slightly tilting the end mirror M, where the red solid circle is the focal point of laser beam, representing a point source S, and the two red open circles are the mirror images of the point source S with respect to the SLM and the BS, respectively. Reflection at SLM then BS gives a virtual image of S at *S*_*B*_, while reflection at BS then the mirror M gives a virtual image of S at *S*_*A*_.

Figure 4 shows the schematic diagram to experimentally demonstrate active control on high-order coherence of two classical point sources. A 780-nm single-mode continuous-wave laser beam was focused to be a small point to mimic a point source. The beam was then input into a Michelson interferometer, where BS was a 50:50 non-polarized beam splitter, M was a reflection mirror and SLM represented a reflection-type phase-only spatial light modulator (HEO 1080P from HOLOEYE Photonics AG, Germany). By slightly tilting the end mirror M to deviate from the perpendicular position (with respect to the incident light on the arm) by a small angle *α*, one could generate two effective point sources *S*_*A*_ and *S*_*B*_ as shown in the inset of Fig. 4. The relative phase difference *φ* between *S*_*A*_ and *S*_*B*_ was controlled through the phase-only spatial light modulator SLM. By adjusting the tilting angle *α*, the distance *d* between *S*_*A*_ and *S*_*B*_ was set to be *d* = 1.43 mm, and the distance between the source plane (determined by *S*_*A*_ and *S*_*B*_) and the detection plane (a CCD camera) was set to be *z* = 136 cm, respectively. The intensity distribution on the detection plane for each relative phase difference *φ* was recorded by the CCD camera with an acquisition time of 1.0 ms. The high-order interference fringes were then calculated through an ensemble average over 1000 frames of the intensity distribution recorded by the CCD camera.

**Figure 5:**
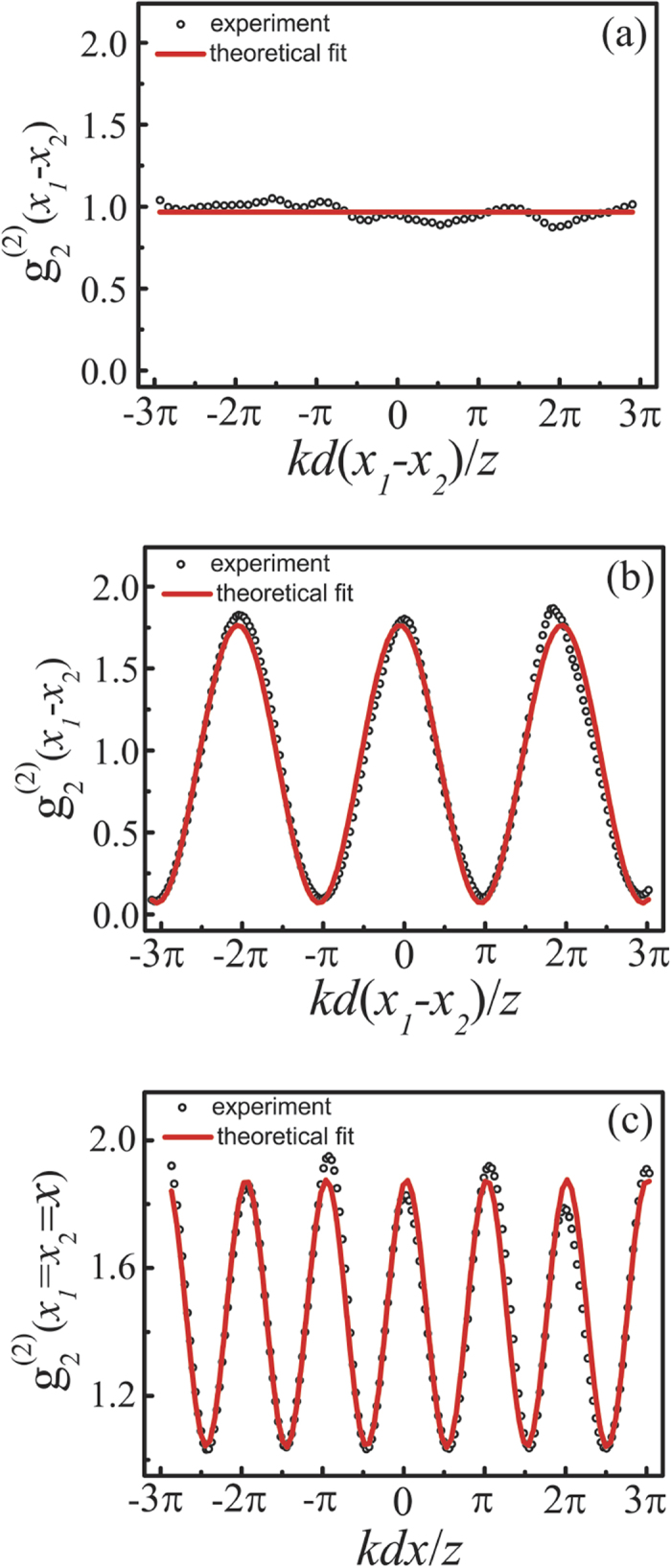
Measured two-photon interference fringes when *φ* varied randomly between {0, *π*} with equal probability 0.5. Here (**a**–**c**) are cases with scanning modes along lines Ia, Ib and II, respectively, in Fig. 2(a). The red solid curves are theoretical fit based on Eq. (2) in different scanning modes.

Figure 5 shows the measured two-photon interference fringes with different position and scanning mode of detectors when the relative phase difference *φ* varied randomly between {0, *π*} with equal probability 0.5. Figure 5(a) corresponds to the case when one detector was fixed while the other detector was scanned along line Ia in Fig. 2(a), no interference fringe was observed in this scanning mode. Figure 5(b) is the case when one fixed one detector (but at different position from that in Fig. 5(a)) and scanned the other detector along line Ib in Fig. 2(a). One sees that two-photon interference fringes with a visibility of 98%, which is close to the theoretically predicted 100% (see Eq. (2) and Fig. 2(b)), could be observed in this configuration even with two classical point sources. Furthermore, synchronous position subwavelength two-photon interference fringes with an effective wavelength of *λ*/2 = 390 nm were observed when one scanned the two detectors simultaneously along line II in Fig. 2(a), in good accordance with the theoretical prediction by Eq. (2).

**Figure 6:**
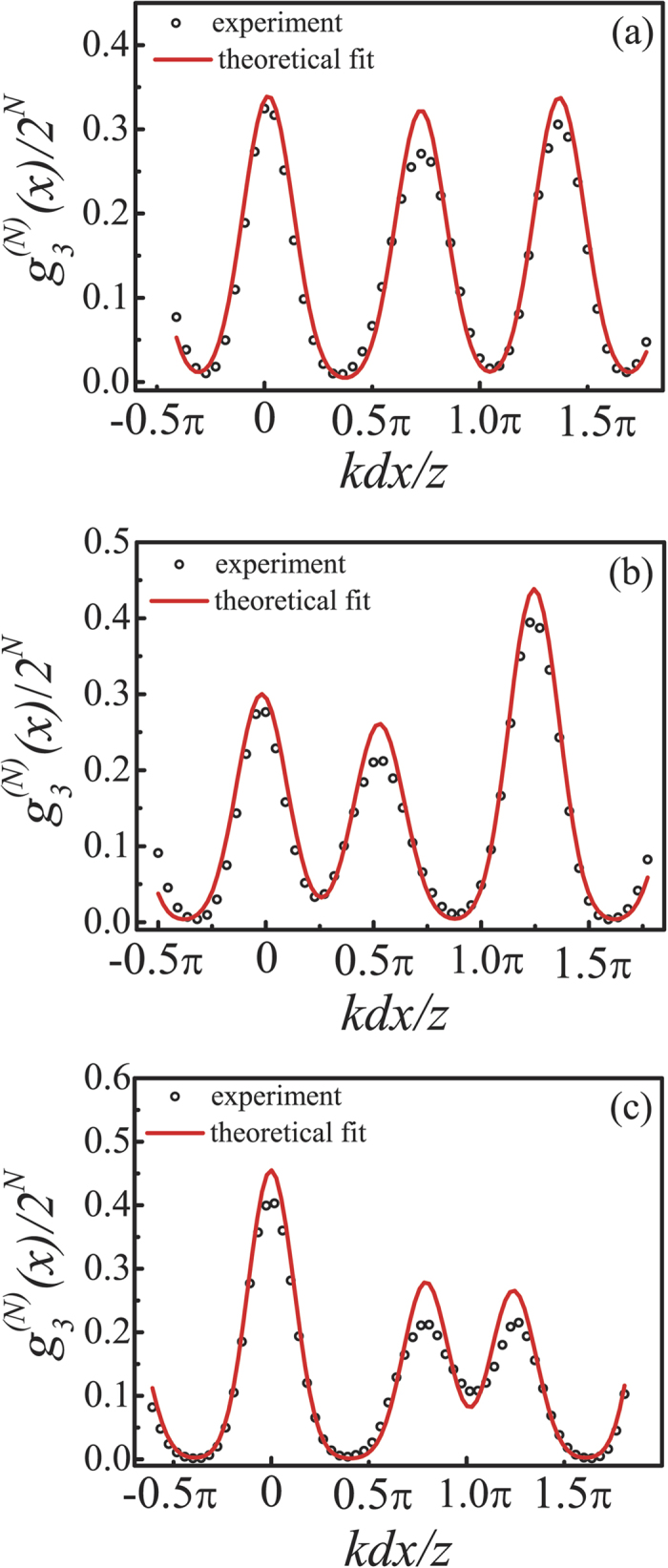
Measured synchronous position high-order interference fringes when *φ* varied randomly among 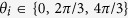 with equal probability 1/3 (**a**), 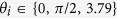 with 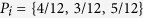 (**b**) and 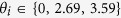 with 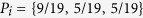 (**c**), respectively. Here *N* = 15 in all cases. The red solid curves are theoretical fit according to Eq. (6) with {*P*_*i*_, *θ*_*i*_} being the free fitting parameters.

More interestingly, synchronous position subwavelength interference fringes with an effective wavelength of *λ*/3 = 260 nm were generated when the relative phase difference *φ* varied randomly among {0, 2*π*/3, 4*π*/3} with equal probability *P*_1_ = *P*_2_ = *P*_3_ = 1/3, as shown in Fig. 6(a). As predicted theoretically by Eq. (6), the fringe pattern can be actively controlled simply by adjusting the critical value *θ*_*i*_ and its probability *P*_*i*_ as long as Eq. (5) is satisfied. Figure 6(b,c) show two additional cases where 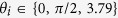 with *P*_*i*_ = {4/12, 3/12, 5/12} and 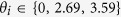 with 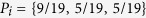, respectively. Note that, just as the case in Fig. 3, the vertical axes in Fig. 6 are again scaled by a factor 2^*N*^. On the contrary, one can also extract the statistic parameters of the random fluctuation of *φ* from the measured synchronous position high-order interference fringes by fitting the experimental data based on Eq. (6). During the fitting process, we set the statistic parameters {*P*_*i*_, *θ*_*i*_} as free fitting parameters under the restriction 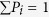, while the experimental values were taken for *k*, *d* and *z*. The extracted parameters were found to be 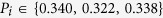 and 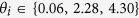 for the case in Fig. 6(a), 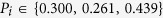 and 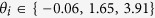 for the case in Fig. 6(b), and 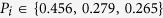 and 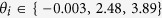 for the case in Fig. 6(c), respectively. As compared to the corresponding theoretical parameters, good agreement with an experimental deviation less than 10% (most of them were within 5%) is achieved, which verified the effectiveness of the proposed method. The slight mismatching is mainly due to the deviation of the relative phase difference *φ* set by SLM from the theoretically required ones because of the imperfect phase linearity and stability of SLM and the vibration instability of the environment.

## Discussion

In general, when the relative phase difference *φ* varies randomly among *M* critical values 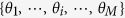, each with a probability 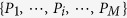, the intensity interference fringes between *S*_*A*_ and *S*_*B*_ will be smeared out under the condition


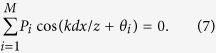


As expected, high-order interference fringes appear in this configuration, and the normalized synchronous position *N*th-order (*N* ≥ *M*) interference fringes can be described by


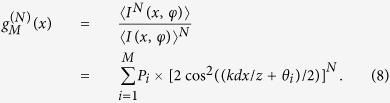


One sees that it is composed of *M* elementary fringes, and the *i*th elementary fringe is characterized by a probability amplitude *P*_*i*_ and a phase shift *θ*_*i*_. Therefore, one can extract the statistic information of the random variation of the relative phase difference *φ* from the *N*th-order interference fringe pattern. Moreover, by equally distributing *θ*_*i*_ within [0, 2*π*) and each with a equal probability of 1/*M*, one can achieve subwavelength interference fringes with an effective wavelength of *λ*/*M*.

It is worth noting that the mechanism to generate subwavelength interference here is completely different from that based on the concept of photonic de Broglie wavelength^6^,^15^, where entangled *M*-photon source is required and *M*-photon correlation measurement will be performed. In contrast, two classical point sources with specially designed random phase fluctuation are used and *N*-photon correlation measurement (*N* ≥ *M*) are performed in our case. The subwavelength interference demonstrated here is also different from those with chaotic thermal light sources, in which the subwavelength interference was realized in a configuration with respect to the position separations among different detectors, *i.e.*, the detectors are located symmetrically and scanning in opposite directions^35^,^36^,^37^,^38^,^39^,^40^,^41^,^42^. In our case, the synchronous position subwavelength interference is realized when the relative phase difference *φ* between two incoherent point sources changes randomly only among several discrete critical values 2*mπ*/*M* 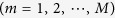 with equal probability and all detectors are at the same spatial position and scanning in the same direction. One may also note that there will be no interference fringes at all with thermal light sources in the synchronous position scanning scheme^39^. In addition, we realized the second-order interference fringes with respect to position separation of detectors (*x*_1_ − *x*_2_) with a 100% visibility (see Figs 2(b) and 5(b)), while the visibility of the second-order interference fringes is always less than 50% with thermal light^35^,^36^,^37^,^38^,^39^,^40^,^41^,^42^.

Oppel *et al.*^17^ reported the (*N* − 1)th-fold subwavelength interference with thermal light through *N*th-order correlation measurement by putting the (*N* − 1) detectors at the so-called magic angles, and Cao *et al.*^32^ reported similar (*N* − 1)th-fold subwavelength interference in the intensity distribution by setting the (*N* − 1) light sources at special magic positions. Although the magic angles in the works by Oppel *et al.* and Cao *et al.* also satisfy Eq. (7) in our work, we would like to point out that the mechanisms to realize subwavelength interference in these two works are completely different from ours. In the works by Oppel *et al.* and Cao *et al.*, the subwavelength interference relies on the suppression of the low spatial frequencies of the sources by setting either the detectors or the light sources at the magic positions. However, in our case the subwavelength interference is realized by increasing the spatial frequency of the interference fringes through control on the statistic behavior of the relative phase difference *φ* between two interfering light sources. Because of the different mechanisms, the properties of the resulting subwavelength interference are also very different. For example, for incoherent light sources, the visibility of the subwavelength interference fringes decreases with the increase of correlation order *N* in the works by Oppel *et al.* and Cao *et al.*, while the visibility increases with the increase of correlation order *N* in our case (see Figs 3 and 6(a) with *N* = 15 as compared to Figs 2(c) and 5(c) with *N* = 2). Also, there is no subwavelength interference fringe in the *N* = 2 case in the works by Oppel *et al.* and Cao *et al.* However, we can realize subwavelength interference with an effective wavelength half of that of the original light sources even in the *N* = 2 case (see Figs 2(c) and 5(c)). Furthermore, the subwavelength interference fringes reported by Oppel *et al.* and Cao *et al.* were realized in the configuration when detectors or light sources were at different positions, that is, the demonstrated subwavelength interference is with respect to the position difference among different detectors^17^ or in the first-order intensity distribution^32^, respectively. While our subwavelength interference fringes are the synchronous position one, *i.e.*, all detectors are exactly at the same spatial position and scanning in the same direction synchronously, which is of practical importance for applications such as optical lithography^43^.

In summary, we showed that it is possible to actively control the high-order coherence of two classical point sources simply by designing the statistic fluctuation of the relative phase difference between these two point sources, indicating that the optical phase difference also plays an important role in the high-order optical coherence. Therefore, by designing the statistic fluctuation of the relative phase difference between two classical point sources, we demonstrated synchronous position subwavelength interference with an effective wavelength much shorter than the original one of the point sources, which may have potential applications in optical lithography and holography with high spatial resolution. Furthermore, the use of classical light with much high intensity in our configuration may overcome the limitation of weak intensity with entangled quantum light source in quantum optical lithography. In addition, we found that the synchronous position high-order interference fringe pattern is actually a fingerprint of the statistic fluctuation of the relative phase difference between two point sources, therefore, one can characterize the statistic distribution of the relative optical phase between two first-order incoherent light sources simply by measuring their synchronous position high-order interference fringe pattern, which may be useful for optical metrology of random processes.

## Methods

### A geometric solution to Eq. (5) and Eq. (7)

**Figure 7:**
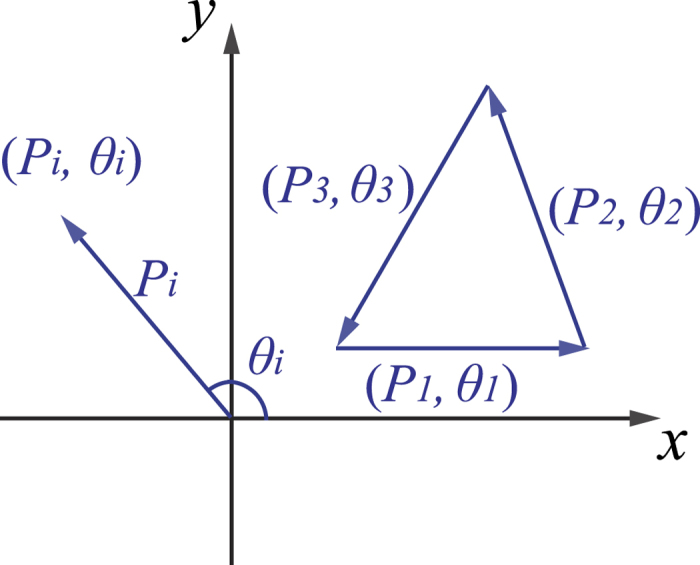
Vector (*P*_*i*_, *θ*_*i*_) represented in an arbitrary Cartesian coordinate system with x- and y-axis as its two orthogonal axes and the closed vectorial triangle constructed by three vectors (*P*_*i*_, *θ*_*i*_) (*i* = 1, 2, 3).

To find out the solution of Eq. (5), one can turn to a more intuitive geometric way, i.e., the vectorial triangle configuration. If one constructs a vector (*P*_*i*_, *θ*_*i*_) with its module and argument being *P*_*i*_ and *θ*_*i*_, respectively, the requirement


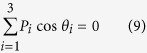


can be met when a closed vectorial triangle is constructed by three vectors (*P*_*i*_, *θ*_*i*_), as shown in Fig. 7. Note that Eq. (9) is also satisfied by rotating each vector (*P*_*i*_, *θ*_*i*_) with the same arbitrary angle *θ*_0_, i.e.,


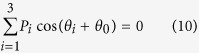


which is exactly the same as Eq. (5) when one sets *θ*_0_ = *kdx*/*z*. Therefore, one can conclude that once the three vectors (*P*_*i*_, *θ*_*i*_) construct a closed vectorial triangle, Eq. (5) is satisfied and the intensity interference fringes between *S*_*A*_ and *S*_*B*_ will be washed out.

To generalize, for arbitrary *M* vectors (*P*_*i*_, *θ*_*i*_), Eq. (7) is satisfied when these *M* vectors form a closed structure. In this geometric viewpoint, for the extreme case of two fully independent point sources *S*_*A*_ and *S*_*B*_ with the relative phase difference *φ* distributed randomly and linearly within [0, 2*π*), the corresponding closed vectorial structure would actually approach to a circle.

## Additional Information

**How to cite this article**: Hong, P. *et al.* Active control on high-order coherence and statistic characterization on random phase fluctuation of two classical point sources. *Sci. Rep.* **6**, 23614; doi: 10.1038/srep23614 (2016).
